# A rare association of chronic lymphocytic leukemia with c-ANCA-positive Wegener’s granulomatosis: a case report

**DOI:** 10.1186/s12957-016-0901-x

**Published:** 2016-05-16

**Authors:** Victoria Bîrluţiu, Elena Cristina Rezi, Rares Mircea Bîrluţiu, Ioan Sorin Zaharie

**Affiliations:** Faculty of Medicine, Lucian Blaga University of Sibiu, Infectious Diseases Clinic - Academic Emergency Hospital Sibiu, Str. Lucian Blaga, Nr. 2A, Sibiu, 550169 Romania; European Hospital Polisano, Sibiu, Str. Izvorului nr. 1A, Sibiu, 550172 Romania; Faculty of Medicine, Lucian Blaga University of Sibiu, Spitalul Clinic de Ortopedie-Traumatologie si TBC Osteoarticular “Foisor” Bucuresti, Str. Lucian Blaga, Nr. 2A, Sibiu, 550169 Romania; Department of Pathology, Academic Emergency Hospital Sibiu, B-dul Corneliu Coposu nr.2-4, Cod: 550245, Sibiu, Romania

**Keywords:** Wegener’s granulomatosis, Chronic lymphocytic leukemia, c-ANCA positive

## Abstract

**Background:**

Wegener’s granulomatosis is a systemic vasculitis of the small- and medium-sized vessels, produced by the action of ANCA, which involves the respiratory tract, kidneys, and eyes, with a potential for lethal evolution in the first year after diagnosis. Its association with chronic lymphocytic leukemia is rarely described in the literature, and it may be difficult to diagnose and to treat this association.

**Case presentation:**

We present the case of a 73-year-old Caucasian patient, a rare case in which Wegener’s granulomatosis is associated in a patient with chronic lymphocytic leukemia, who is admitted in the Infectious Disease Department for fever, diplopia, headache, purulent and hemorrhagic nasal secretions, intense asthenia, and weight loss. The patient had associated eyelid edema; scleritis; chemosis; subconjunctival hemorrhage at the left eye; swelling of the left region of the eyehole, of the base of the nasal pyramid, and of the left zygomatic region; anterior nasal bleeding; pustulous non-itching lesions at the cervical region and posterior thorax; enlarged bilateral axillary lymph nodes; hepatomegaly; and moderate splenomegaly. During the surgical treatment of the pansinusitis, a biopsy from the tissue is taken; the biopsy fragments of the nasal mucosa pleads for Wegener’s granulomatosis. The c-ANCA were positive. The patient’s evolution was favorable under treatment with meropenem, teicoplanin, fluconazole, transfusions of platelet concentrates, and methylprednisolone.

**Conclusions:**

The real dimension of the association between chronic lymphocytic leukemia and Wegener’s granulomatosis is not known; it may be useful to evaluate the vasculitis by testing ANCA routinely in patients with chronic lymphocytic leukemia and by histopathological examinations of the lesions.

## Background

Wegener’s granulomatosis is a systemic vasculitis of the small- and medium-sized vessels or a necrotizing vasculopathy, produced by the action of antineutrophil cytoplasmic antibodies (ANCA). In Wegener’s granulomatosis, 95 % of the lesions are present in the upper and inferior respiratory tract and sometimes associated with renal or ocular lesions [1]. There are described infiltrating pulmonary lesions, with centrilobular disposition, with a “matte glass” aspect, and hemorrhage lesions, with or without pleural effusion; rarely, a bronchitis or tracheal stenosis may appear. The upper respiratory tract lesions have—as clinical manifestations—rhinorrhea, with mucous, purulent, hemorrhagic secretions; the perforation of the nasal septum; medium otitis; and sensorineural hearing loss. The lesions involving the eye may include conjunctivitis, episcleritis, and optical nerve vasculitis. At the oral cavity level, there may appear bone destruction, responsible for the loss of the teeth or for ulcerative mucosal lesions. Without an immunosuppressive treatment, the disease evolution is associated with a high mortality rate, in the first year following the first signs.

## Case presentation

**Fig. 1:**
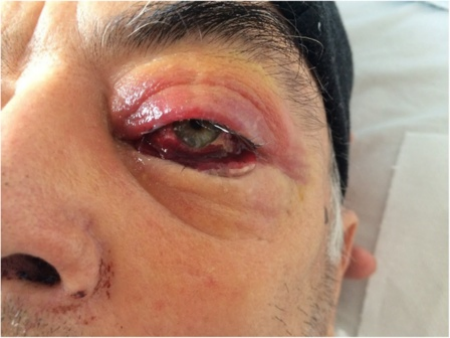
The eye involvement of Wegener’s granulomatosis and signs of an acute epistaxis. Eyelid edema, scleritis, chemosis, and subconjunctival hemorrhage of the left eye

We present the case of a 73-year-old Caucasian patient, diagnosed 8 years ago with chronic lymphocytic leukemia, stage B Binet, who was in treatment with chlorambucil and prednisone, and who presented in the last year an abscessed pneumonia, with *Pseudomonas aeruginosa.* He is admitted in the Infectious Disease Department for fever, diplopia, headache, purulent and hemorrhagic nasal secretions, intense asthenia, weight loss, and symptoms which appeared after a dental extraction which was performed 10 days before admission and for which the patient had received treatment with clarithromycin 500 mg/day. Upon admission, the clinical examination revealed the following: febrile patient 39 °C with altered general status; pallor; eyelid edema; scleritis; chemosis; subconjunctival hemorrhage at the left eye (Fig. 1); swelling of the left region of the eyehole, of the base of the nasal pyramid, and the left zygomatic region; anterior nasal bleeding; pustulous non-itching lesions at the cervical region and posterior thorax; swelling of the bilateral axillary lymph nodes; blood pressure of 110/60 mmHg; heart rate of 85/min; hepatomegaly; and moderate splenomegaly.

From the laboratory tests we performed, the following were significantly altered: leucocytes 24,620–40,660/mm^3^, hemoglobin 11.6–10 g/dl, hematocrit 35.3–31.2 %, neutrophils 10–12 %, lymphocytes 84.3–81 %, platelets 34,000–132,000/mm^3^, erythrocyte sedimentation rate 61 mm/h, fibrinogen level 517.6 mg/dl, fibrin monomers—absent, and C-reactive protein 7.73–192 mg/l. The bacteriological exam of the conjunctiva secretions revealed the following: the Gram coloration indicated the presence of epithelial cells, *hematies*, and Gram-positive flora. Three hemocultures were negative for bacteria and fungi. In the bacteriological exam of the nasal exudates, *Klebsiella pneumoniae* was isolated. The urine sample result was as follows: pH 7, erythrocyte positive, proteins 30 mg/dl, urobilinogen 2 mg/dl, and ascorbic acid 20 mg/dl; the urinary sediment showed frequent yeast and rare leucocytes, and the culture for fungi was positive.

The anterior face sinus radiography showed an almost complete opacity of the left maxillary sinus, opacity of the left ethmoidal cells and of the inferior half of the left frontal sinus (sinus collection), and the hypertrophy of the inferior left nasal cornets.

**Fig. 2:**
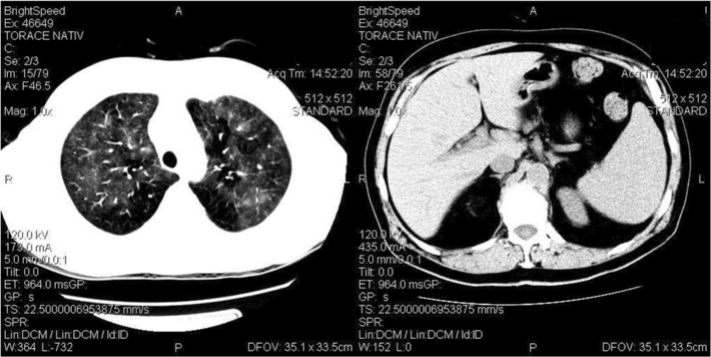
CT scan images. CT images show peripheral and peribronchial patchy ground-glass opacity in both lungs (*left*) and homogenous hepatosplenomegaly (*right*)

The thoracic CT scan showed a bilateral matte glass pulmonary aspect or ground-glass opacity and a dense star-shaped mass in the right inferior lobe, situated in the apical segment, with a diameter of 3.5 cm with extension towards the adjacent pulmonary parenchyma (Fig. 2). The abdominal CT revealed homogenous hepatosplenomegaly.

The otorhinolaryngology specialist considered opportune the surgical treatment of the pansinusitis; during the surgery, a biopsy sample was taken for the histopathological and bacteriological exams. The cultures were positive for *K. pneumoniae* (with sensitivity at fosfomycin, imipenem, and meropenem) and *Enterococcus faecalis* (with sensitivity to ampicillin, ciprofloxacin, clarithromycin, erythromycin, gentamicin, vancomycin, streptomycin, teicoplanin, and rifampicin).

**Fig. 3:**
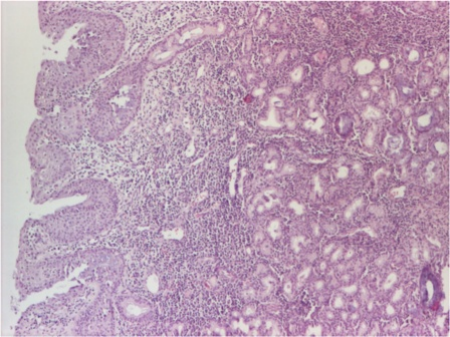
Histopathological aspect. The nasal mucosa has chronic rhinitis-like alterations—thickening of the lining tissue with hyperplasia of the mucous glands and a moderate augmentation of the chronic inflammatory infiltrate. Pavement metaplasia at the surface epithelial level. HE staining, ×40

**Fig. 4:**
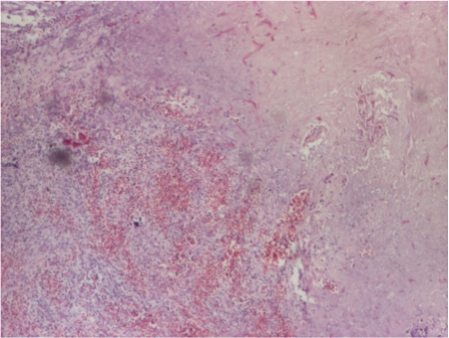
Histopathological aspect. Mucosa with ulceration, necrosis, and hemorrhage. HE staining, ×40

**Fig. 5:**
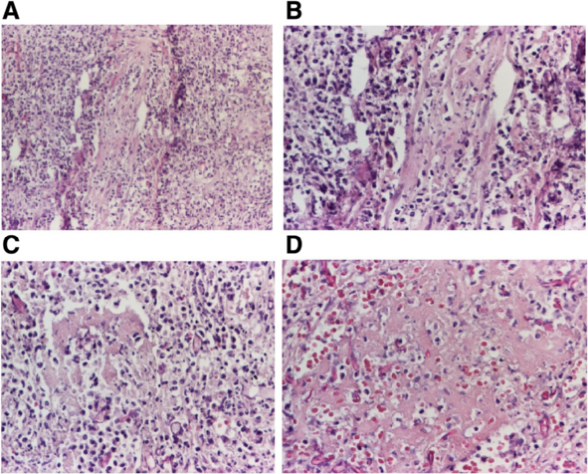
Histopathological aspects. Vasculitis with the presence of polymorphonuclear neutrophils on the vascular wall, with leukocytoclastic aspect (**a** HE staining, ×100; **b** HE staining, ×200) and fibrinous exudate (**c, d** HE staining, ×100). Vasculitis process (caught in a tangential, longitudinal section) with inflammatory destruction of the blood vessel wall with the presence of polymorphonuclear neutrophils on the vascular wall, with leukocytoclastic aspect and fibrinous exudate

**Fig. 6:**
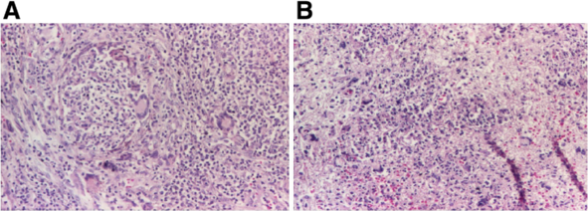
Histopathological aspects. Suppurative granulomas with multinucleated giant cells (**a** HE staining, ×200, **b** HE staining, ×100)

The biopsy fragments of the nasal mucosa revealed an intense lymphoplasmacytic hystiocitary inflammatory infiltrate, with the presence of eosinophils, focal polymorphonuclear cells, giant multinucleate Langhans cells, and vasculitis. The histopathological examination pleads for Wegener’s granulomatosis (Figs. 3, 4, 5, and 6).

Histopathological examination conclusion: The microscopic aspect advocates for a necrotic lesion with vasculitis and gigantocellular reaction aspects, Wegener’s granulomatosis type. The lesions may be in the early stages of evolution, which is why typical granulomas are not present.

The c-ANCAs (cytoplasmic antineutrophil cytoplasmic antibodies) were performed, with a positive result.

The patient’s evolution was favorable under treatment with meropenem, teicoplanin, fluconazole, transfusions of platelet concentrates, and methylprednisolone.

### Discussions

The patient experienced an episode of infectious shock a year before admission in our department, with severe acute respiratory insufficiency, with purulent, hemorrhagic cough; acute renal insufficiency (with creatinine levels of 5 mg/dl); decreasing of the number of leucocytes (from 93,690 to 5100/mm^3^); severe neutropenia (100/mm^3^); and low platelet count (42,000/mm^3^). The thoracic CT scan revealed a nodular opacity of the right inferior lobe, situated in the apical segment, with a tendency towards abscessing, measuring 6/3 cm; imprecise bordered opacities in the left perihilar area; and minimal right pleurisy. The dermatological lesions presented at that moment were interpreted as post-drug-fixed erythematic lesions, being treated with prednisolone 32 mg/day, with the decreasing of the doses and treatment cessation after 6 weeks. The repeated respiratory infections were considered to result from the chronic lymphocytic leukemia, caused by the severe immune deficit, and the case was not investigated for vasculitis, this situation being rarely described in the literature [2, 3]. The association of eyehole cellulites with the rhino sinusal lesions on the same region (Fig. 2) completed the clinical aspect with elements which are related to Wegener’s granulomatosis, confirmed by biopsy and by positive c-ANCAs, a situation which is rarely described in the literature [4, 5] in association with chronic lymphocytic leukemia. The diagnosis of granulomatosis would have not been possible without the pathological anatomical result.

The difference between the manifestations produced by chronic lymphocytic leukemia and the one attributed to Wegener’s granulomatosis was difficult to establish. Retrospectively, we can affirm that the repeated pulmonary manifestations and the renal failure were due to Wegener’s granulomatosis, given the known fact that chronic lymphocytic leukemia usually evolves with membranoproliferative or cryoglobulinemic glomerulonephritis or with amyloidosis [6]. The persistent pulmonary manifestation, proved by CT scan, with a tendency towards abscessing, even though the patient was in clinical remission; the opacity with a matte glass appearance; and the pleural effusion are also associated with Wegener’s granulomatosis [7]. The eye lesions were also associated with all of these, which are described in more than half of the cases with Wegener’s granulomatosis [8]: the inflammatory eyehole disease, scleritis, chemosis, maxillary sinus lesions, and left frontal and ethmoidal lesions, confirmed by biopsy. The severe neutropenia which the patient presented was probably due to the presence of ANCA [3], while the low platelet number was a consequence of the chronic lymphocytic leukemia [9]. Because of the immune deficit (during the evolution of chronic lymphocytic leukemia), hypogammaglobulinemia, complement deficit [10–12], and corticotherapy, the patient presented a high risk of respiratory tract infections with encapsulated bacteria—*K. pneumoniae*, and of sepsis with *P. aeruginosa*, risks which are described in the literature [13]. The patient’s evolution was unfavorable, with the repeating respiratory tract infections and death due to sepsis with *K. pneumoniae*.

## Conclusions

In conclusion, the real dimension of the association between chronic lymphocytic leukemia and Wegener’s granulomatosis is not known; it may be useful to evaluate the vasculitis by testing ANCA routinely in patients with chronic lymphocytic leukemia and pathological anatomical examinations of the lesions.

### Consent information

Written informed consent was obtained from the patient for publication of this case report and any accompanying images. The study was accepted by the Ethics Committee of the hospital, and they encouraged publishing the article. A copy of the written consent is available for review by the Editor-in-Chief of this journal.

### Availability of data and materials

The authors do not wish to share their data; they respect the patient’s rights to privacy and to protect his identity. The authors presented, in the manuscript, all the necessary information about their case report. Raw data regarding our patient is in his admission file, a file that is strictly confidential, without the possibility of publishing raw data from it.
